# Supplementary material for: KIF20A/MKLP2 regulates the division modes of neural progenitor cells during cortical development
Source: Nat Commun. 2018 Jul 13;9:2707. doi: 10.1038/s41467-018-05152-1 (PMC6045631; doi:10.1038/s41467-018-05152-1)
Supplement: Supplementary file 1 — Supplementary Information [file 41467_2018_5152_MOESM1_ESM.pdf]

Supplementary Information

KIF20A/MKLP2 regulates the division modes of neural progenitor cells during cortical development

Geng et al.

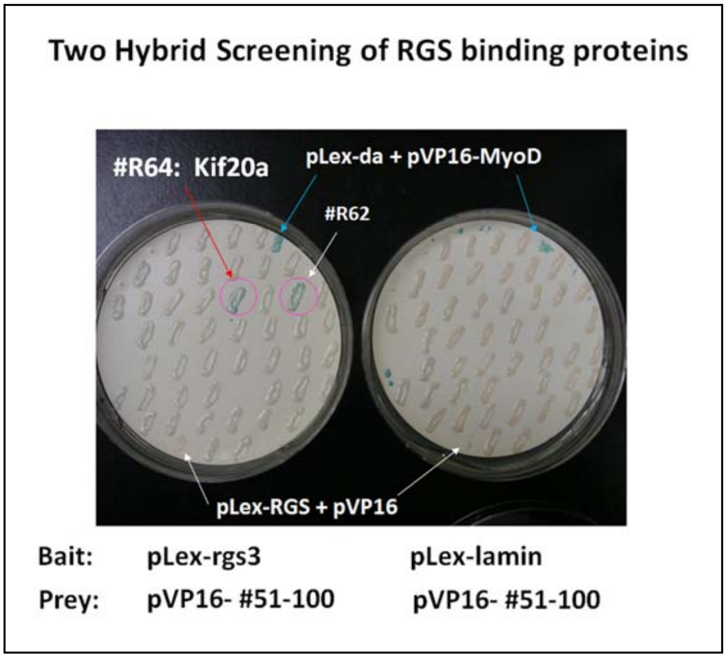

Supplementary Figure 1 Two-hybrid identification of RGS-KIF20A interaction
KIF20A was identified as a candidate interacting protein of RGS3 in two-hybrid screen using either RGS3 full length or the RGS domain alone as bait. The plates of yeast patches showed secondary screen of the primary positive clones #51-100 (pVP16-vector-based library cDNAs) by mating with pLex-vector-based RGS3 bait (plate on the left) or Lamin control bait (plate on the right). #R64 clone (Kif20a cDNA fragment) reproduced positive interaction with RGS3 as indicated by blue color of Xgal detection.
pLex-Da + pVP16-MyoD mating (blue arrows) was used as a positive control for protein-protein interaction. pLex-RGS3 + pVP16 mating (white arrows) was a negative control.

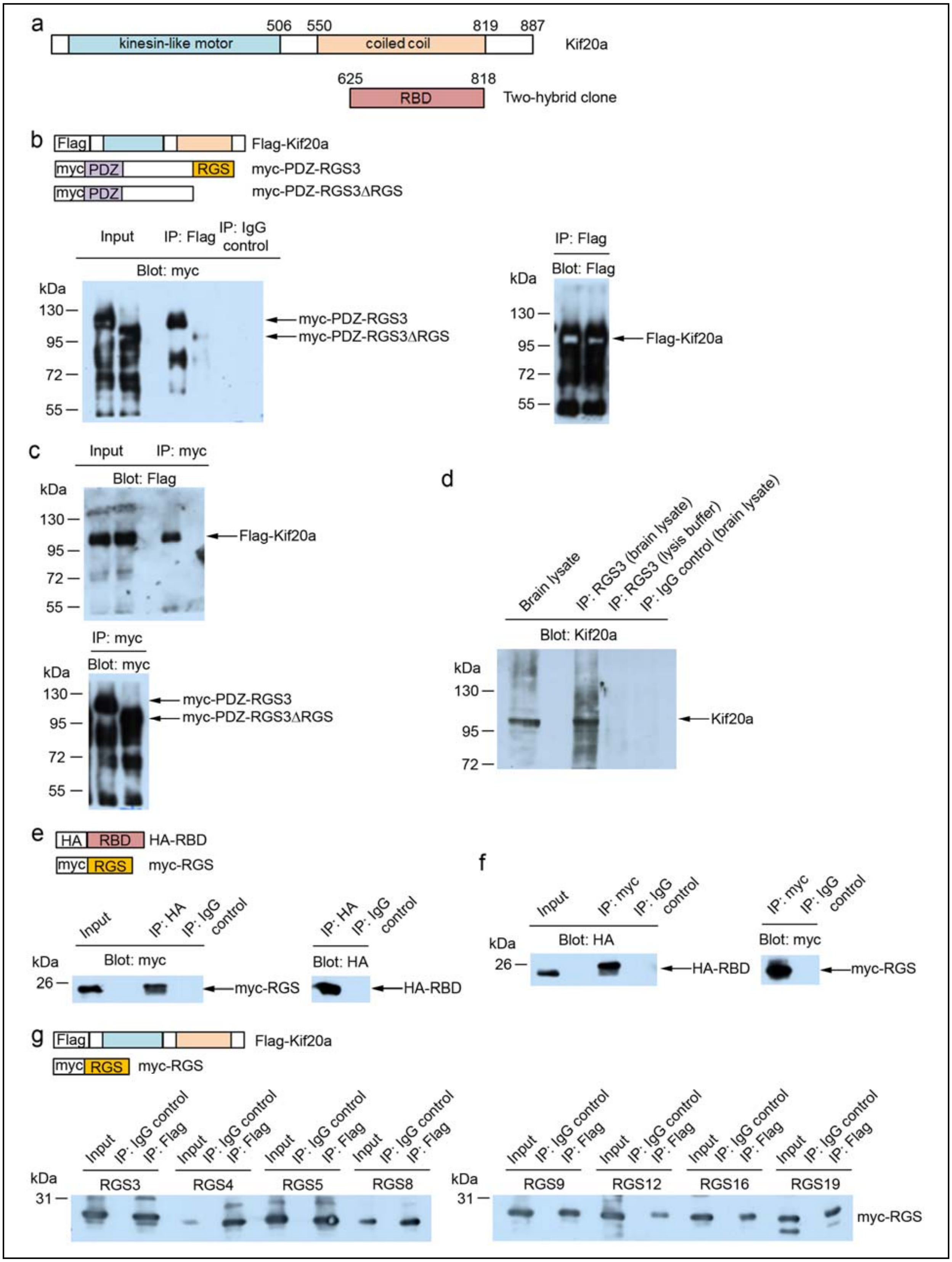
a
506
550
819
887
kinesin-like motor
coiled coil
Kif20a
625
818
RBD
Two-hybrid clone
b
Flag-Kif20a
myc-PDZ-RGS3
myc-PDZ-RGS3ΔRGS
Input
IP: Flag
IP: IgG control
Blot: myc
kDa
myc-PDZ-RGS3
myc-PDZ-RGS3ΔRGS
Blot: Flag
Flag-Kif20a
c
IP: myc
d
Brain lysate
IP: RGS3 (brain lysate)
IP: RGS3 (lysis buffer)
IP: IgG control (brain lysate)
Blot: Kif20a
Kif20a
e
HA-RBD
myc-RGS
IP: HA
Blot: HA
f
g
RGS3
RGS4
RGS5
RGS8
RGS9
RGS12
RGS16
RGS19

Supplementary Figure 2 KIF20A binds to the RGS domain of RGS3

a. Domain structure of mouse KIF20A and the yeast two-hybrid clone identified in the screening. RBD, RGS binding domain.

b and c. Reciprocal co-immunoprecipitation (co-IP) of Flag-KIF20A with Myc-PDZ-RGS3 or Myc-PDZ-RGS3ΔRGS (with RGS domain deleted). Expression plasmids were transfected into Hek293 cells. Co-expressed proteins in cell lysates were precipitated for KIF20A (rabbit-anti-Flag) or PDZ-RGS3 (mouse-anti-Myc) followed by Western blot for the other protein. Left panel in B or upper panel in C showed co-IP of the two full-length proteins but not the mutant RGS3 protein with RGS domain removed. Right panel in B or lower panel in C was a control showing the initial IP worked well for anti-Flag (B) or anti-Myc (C). IgG control was from the same species as anti-Flag antibody.

d. Co-IP of endogenous KIF20A and RGS3 proteins. Cell lysates were made from the E15.5 mouse brains and proteins were immunoprecipitated with rabbit-anti-RGS3 antibody or control rabbit IgG followed by Western blot for KIF20A using a goat-anti-KIF20A antibody. In another control lane, anti-RGS3 antibody was precipitated in lysis buffer instead of brain lysate.

e and f. Reciprocal co-IP of the RBD (RGS-binding domain) of KIF20A and the RGS domain of RGS3.

g. Co-IP of KIF20A with RGS domains from multiple subclasses of the RGS family.

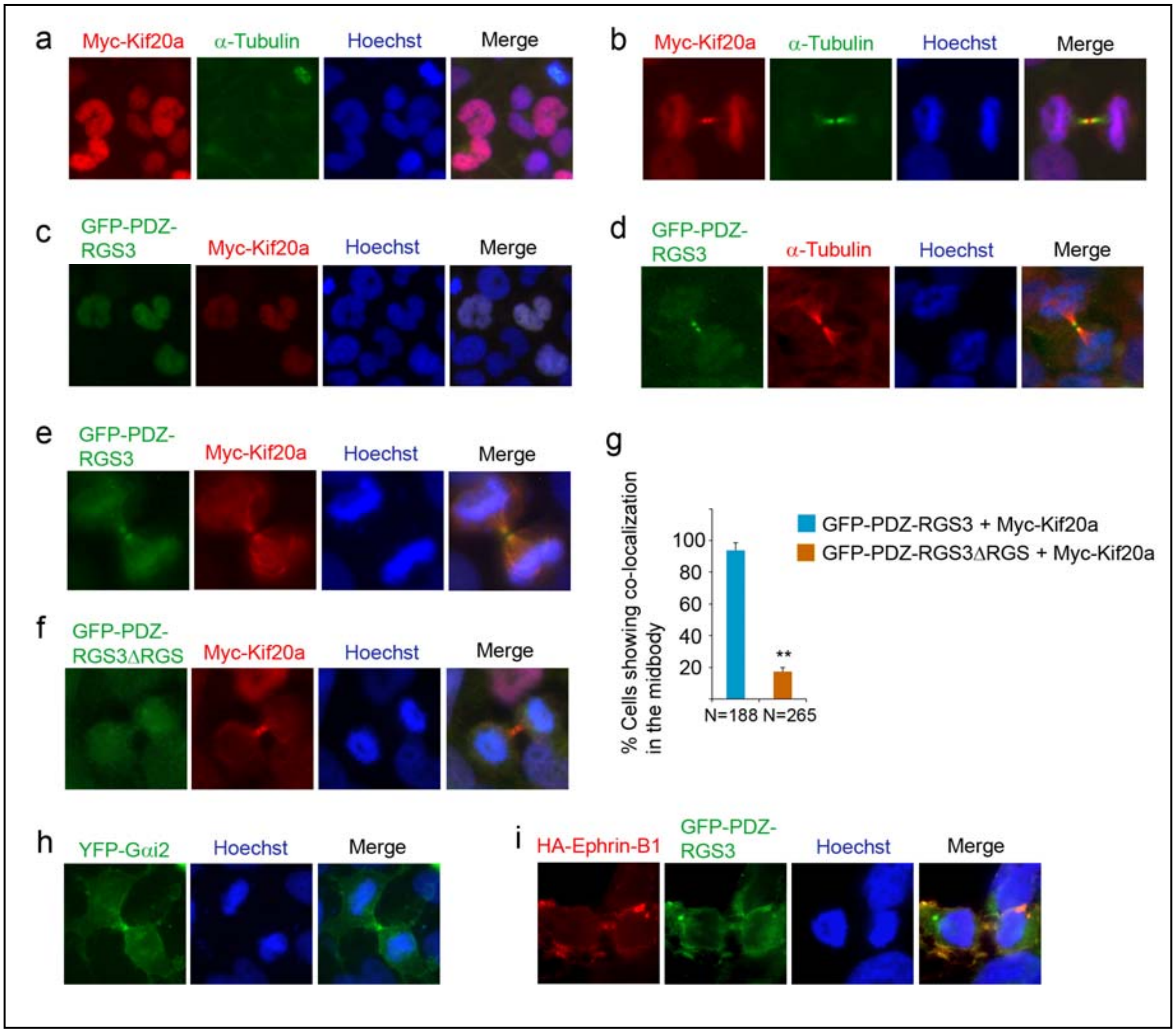

Supplementary Figure 3 KIF20A and PDZ-RGS3 co-localize in the intercellular bridge in dividing cells

a, b. Subcellular localization Myc-KIF20A was observed in an inducible CHP100 cell line for Myc-KIF20A expression. In doxycycline induced cells, Myc-KIF20A was seen in mitotic cytoplasm in early mitosis (**a**) and at midbody in telophase (**b**).

c-e. When transfected into the inducible cell line, GFP-PDZ-RGS3 was seen co-localized with Myc-KIF20A in mitotic cytoplasm (**c**) and at midbody in telophase (**d, e**).

f. Co-localization of KIF20A and PDZ-RGS3 was dependent on the RGS domain, because an RGS domain deletion mutant did not show the pattern of midbody enrichment when co-expressed with Myc-KIF20A.

g. Quantification comparing between GFP-PDZ-RGS3 and the RGS domain deletion mutant for co-localization at midbody with Myc-KIF20A. Of the cases where the deletion mutant was scored positive for co-localization at midbody with Myc-KIF20A, the level of GFP signal was also significantly reduced comparing to that of the wild-type GFP-PDZ-RGS3. **$P<0.01$ (Student's t-test).

h. Expression plasmid of YFP-Gαi2 was transfected into CHP100 cells. YFP signal could be seen enriched in the intercellular bridge of dividing cells.

i. Expression plasmids of HA-Ephrin-B1 and GFP-PDZ-RGS3 were co-transfected into CHP100 cells. GFP signal and anti-HA staining signal could be found co-expressed in the intercellular bridge of telophase cells.

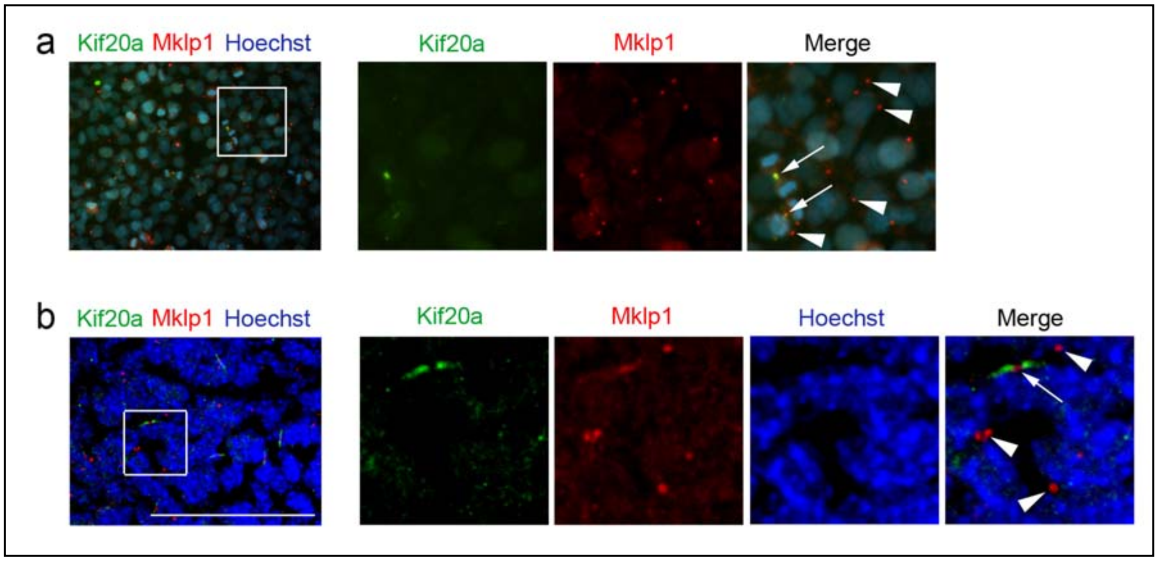

Supplementary Figure 4 KIF20A is not detected in Midbody Ring/Remnant (MR) after midbody release

a. Cultured CHP100 cells were co-stained for endogenous expression of KIF20A (green) and MKLP1 (red). KIF20A and MKLP1 were both present in the intercellular bridge/midbody with KIF20A flanking the MKLP1 ring (indicated by white arrows). KIF20A did not appear to be present in the structures resembling midbody ring/ remnant (MR) marked by MKLP1 (indicated by white arrowheads).

b. En-face view of a whole-mount anti-KIF20A and anti-MKLP1 co-staining with E14.5 cortices showed KIF20A and MKLP1 were both present in the intact intercellular bridge/midbody (indicated by white arrow), but KIF20A staining was not associated with many individual MKLP1-positive speckles which were likely midbody remnants yielded by cell cleavage (indicated by white arrowheads). Scale bar represents 100 μm.

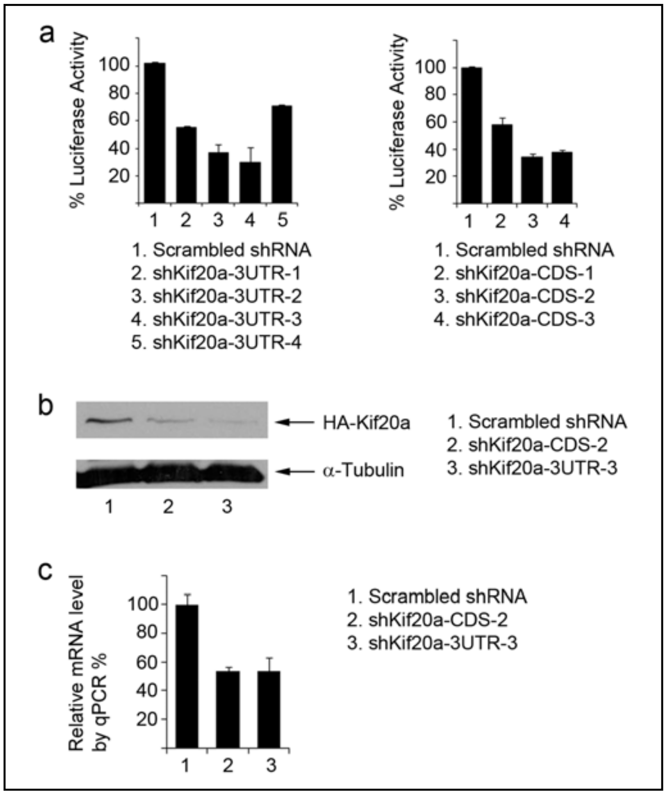

Supplementary Figure 5 Screening of KIF20A shRNAs

a. Screen of shRNAs by luciferase assay. shRNAs and their cDNA target in psi-CHECK Vector (Promega, C8021) were co-transfected into HEK293 cells in triplicates; 48 hours later, the firefly and *Renilla* Luciferase values were determined with Promega's Dual-Luciferase® Reporter Assay System (E1910). The final inhibition unit was the normalized value (Renilla/Firefly). Candidate shRNAs (four against KIF20A-3UTR and three against KIF20A-CDS) were tested with this assay. Data are mean ± S.D.

b. Validation of shRNAs in transfected Hek cells Western Blot. shKIF20A-CDS#2 and shKIF20A-3UTR#3 (based on luciferase screen) were further tested by co-transfection with an expression plasmid of HA-KIF20A into HEK293 cells. Cell extracts were prepared 48 hours after transfection for Western Blot. Significant knockdown effect by the two shRNAs could be detected with α-tubulin as a loading control. Sequences of shKIF20A-CDS#2 and shKIF20A-3UTR#3 were described in Experimental Procedures.

c. Validation of shRNA efficiency in electroporated cortical cells. shRNA plasmids were electroporated into the cortices at E13.5, brains were collected at E15.5 and GFP positive cells were isolated by fluorescence-activated cell sorting (FACS). qPCR was performed on reverse transcribed RNA samples made from the sorted cells in triplicates. Data are mean ± S.D.

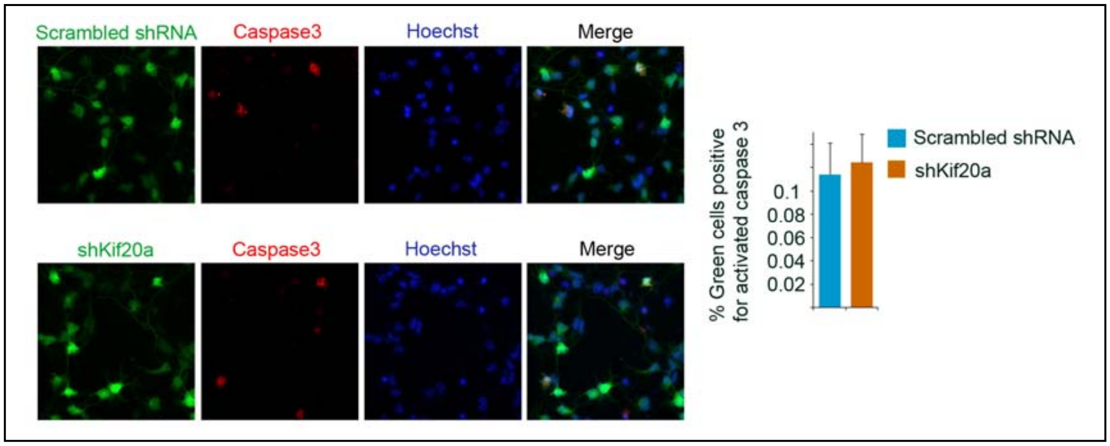

Supplementary Figure 6 Status of apoptosis in cortical cells expressing shKif20a
shKif20a or control shRNA was introduced into dissociated cells derived from the E13.5 mouse cortices by lentiviral expression. After culturing for two days post infection, the cells were fixed for staining with antibody against activated caspase 3. Percentages of green cells that are positive for activated caspase 3 were scored. Very few cortical cells showed signs of apoptosis and no obvious difference was observed between shKif20a- or control shRNA-expressing cells. P=0.49 (Student's t-test).

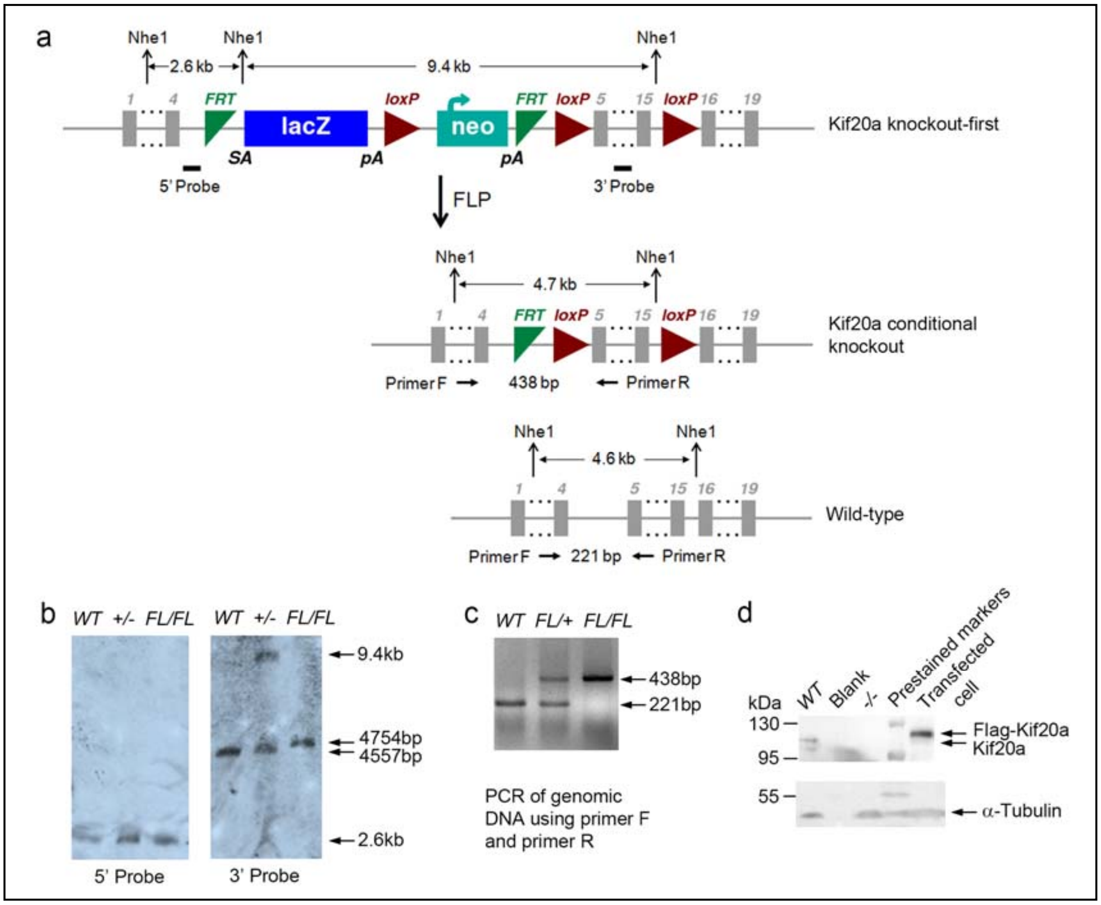

Supplementary Figure 7 Generation of *Kif20a* knockout-first (germline) and conditional mice

a. Illustration of targeting (based on International Mouse Phenotyping Consortium) and validation strategy. Two independent probes were designed for Southern blot using genomic DNAs digested with Nhe1 enzyme. Removal of the LacZ and Neomycin cassette by flippase (FLP) converted knockout-first strain to conditional knockout. PCR primers for genotyping conditional and wild-type alleles were indicated.

b. Southern blot on genomic DNAs from embryos of different genotypes confirmed the genetic targeting.

c. PCR using primers described in (**a)** could distinguish between the conditional and wild-type alleles.

d. Whole cell extracts made from WT (wild-type) or -/- (homozygous germline knockout) forebrains of E12.5 littermates were probed with an anti-KIF20A antibody. Flag-KIF20A expressed from HEK293 cells was used as a control for KIF20A detection and α-Tubulin was used as a loading control for brain cell extracts.

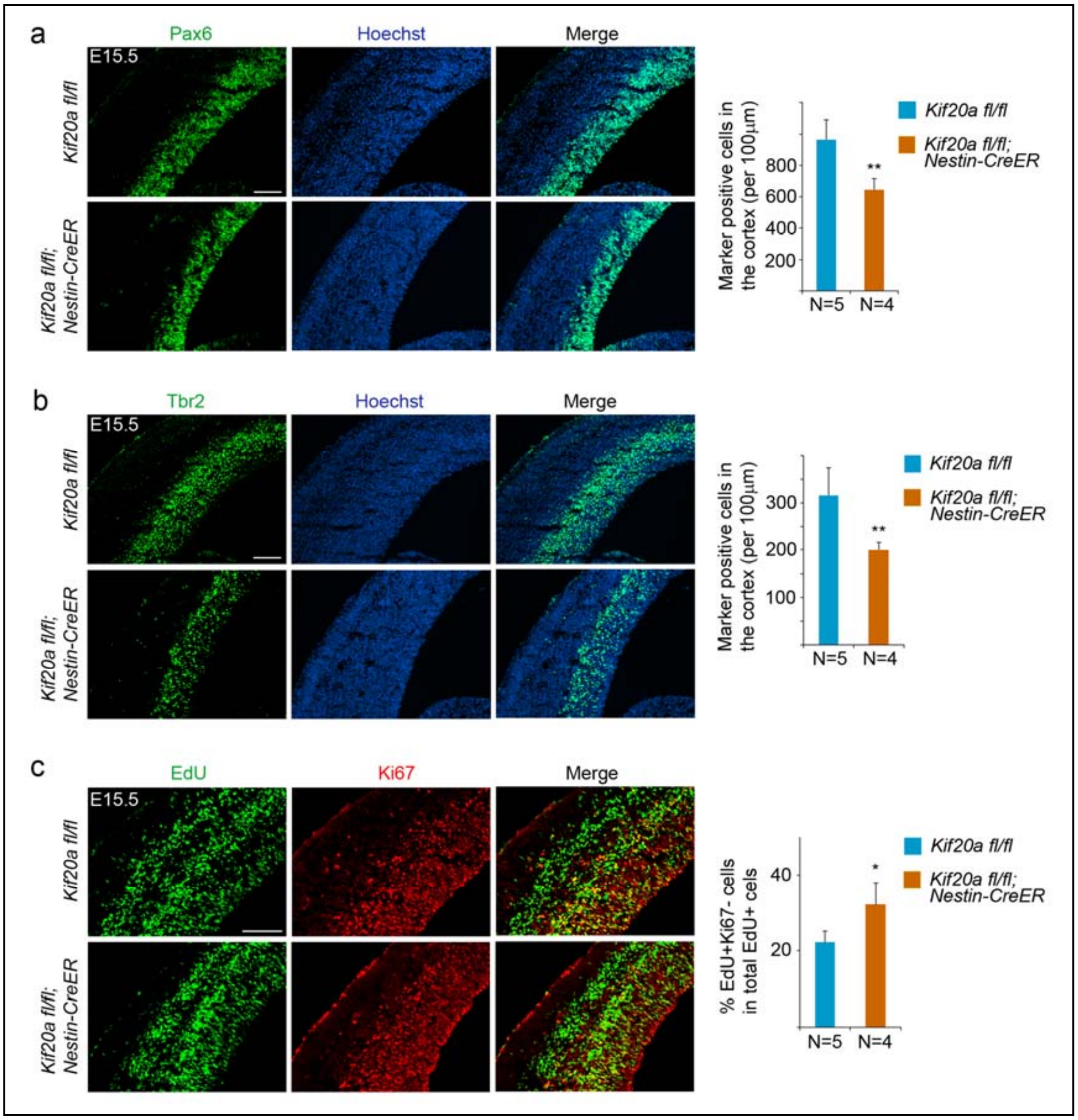

Supplementary Figure 8 Inducible knockout of *Kif20a* causes a loss of cortical NPCs

a. Inducible *Kif20a* knockout caused a loss of Pax6$^+$ radial glial cells. Tamoxifen was administered at E9.5 and E10.5 consecutively. **P<0.01. Scale bar represents 100 μm.

b. Inducible *Kif20a* knockout brains had fewer Tbr2$^+$ intermediate progenitor cells. **P<0.01. Scale bar represents 100 μm.

c. Inducible *Kif20a* knockout caused early cell cycle exit in cortical neural progenitor cells. EdU was injected at E14.5 and the labeled brains were collected for analyses 24 hours later. * P<0.05. Scale bar represents 100 μm.

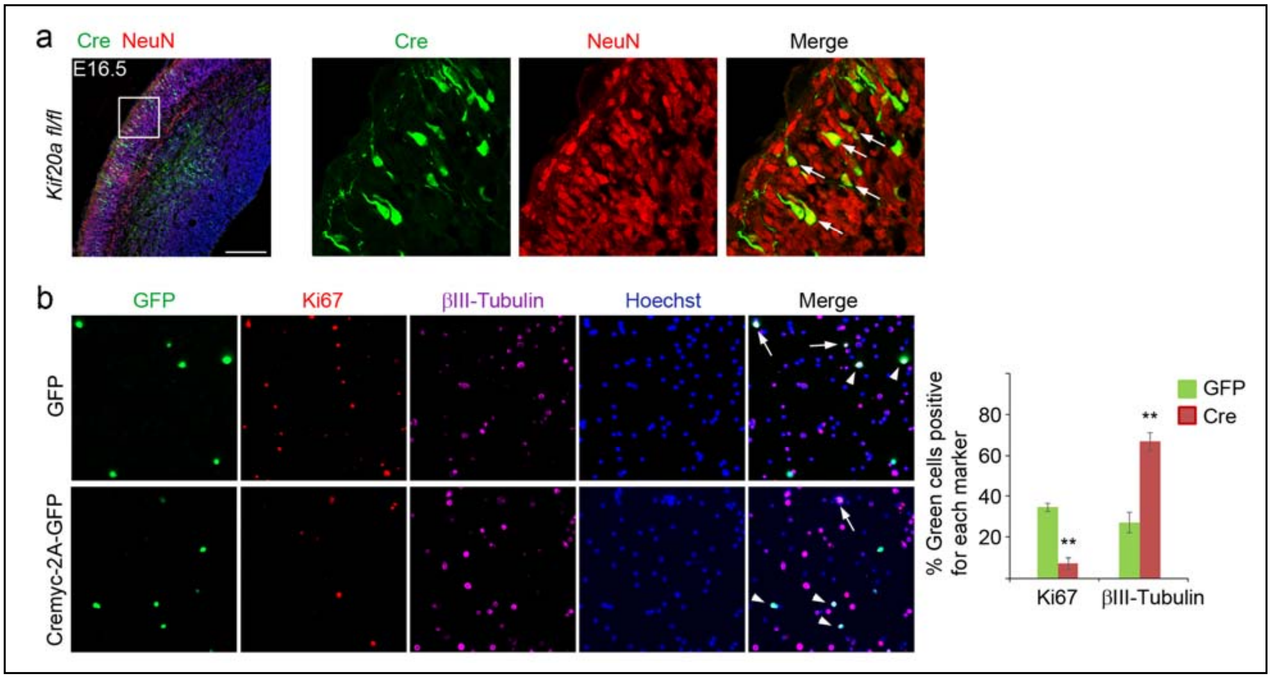

Supplementary Figure 9 Early neuronal differentiation in IUE-mediated conditional knockout of *Kif20a* in the cortex

a. In the cortices of *KIF20A*$^{fl/fl}$ conditional knockout mice, Cre-expressing cells (GFP^{+} cells) that have migrated into the CP were positive for NeuN, suggesting early differentiation into neurons due to *Kif20a* knockout. Arrows indicated examples of GFP^{+}NeuN^{+} cells. Scale bar represents 100 μm.

b. CAG-Cremyc-2A-GFP or control GFP plasmids were introduced into the cortices of *KIF20A*$^{fl/fl}$ conditional knockout mice by IUE at E13.5. Dissociated cortical cells from electroporated brains (collected at E16.5) were prepared, plated down on poly-D-lysine-coated coverslip and cultured for 2 hours. Expression of proliferating cell marker Ki67 or neuronal marker βIII-tubulin was then examined. Arrows and arrowheads indicated examples of Ki67^{+} and βIII-tubulin^{+} cells, respectively. ** P<0.01. Error bars represent SD.

a

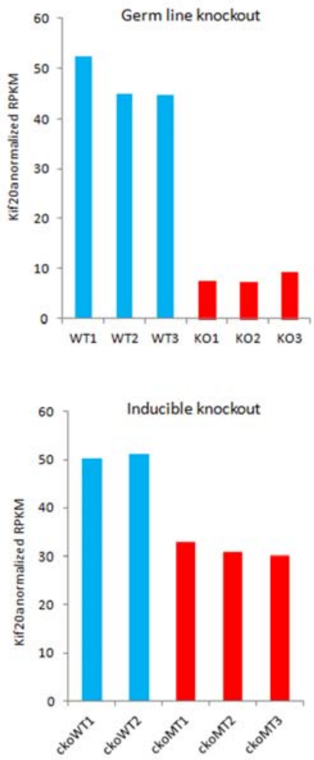

b

	Symbol	WT	KO	Log2FC	P-value
		Germ line knockout			
Down-regulated gene	Fabp7	542.59	295.38	-0.80	0.00405
	Ccnd2	384.52	207.34	-0.92	0.007972
	Snn	71.41	38.65	-0.98	0.000762
	Cycs	56.69	36.91	-0.65	0.020007
	Kif20a	47.41	8.02	-2.54	1.68E-23
	Arf2	40.94	27.50	-0.63	0.011835
Up-regulated gene	Eno1b	301.54	678.89	1.05	0.004553
	Eno1	240.63	562.79	1.08	0.003438
	Ldha	241.85	524.52	1.03	0.002327
	Pgk1	230.93	431.48	0.85	0.008759
	Tpi1	181.48	371.02	0.94	0.004883
	Aldoa	96.27	331.58	1.53	0.000171
	Igfbp2	126.07	313.44	1.18	0.00111
	Fam162a	93.94	207.62	1.01	0.001415
	Ddit4	28.86	191.63	2.04	0.001568
	Slc2a1	31.23	152.18	1.90	3.06E-05
		Inducible knockout			
	Symbol	ckoWT	ckoMT	Log2FC	P-value
Down-regulated gene	Kif20a	50.73	31.31	-0.70	3.38E-17
	Rspo1	39.61	23.07	-0.84	0.000228
	Wnt8b	33.67	23.09	-0.60	0.006169
	Rspo2	22.91	13.95	-0.81	0.005882
	Kdm6a	19.54	12.64	-0.63	1.50E-11
	Foxj1	16.98	9.60	-0.84	2.83E-06
	Slc6a15	15.61	10.36	-0.61	0.000111
	Clic6	12.63	8.43	-0.61	0.001301
	Dkk3	9.20	5.94	-0.65	0.000138
	Nfatc4	9.22	5.87	-0.68	0.000167
Up-regulated gene	Fgf15	34.36	51.54	0.59	8.77E-06
	Eif2s3y	0.00	21.58	9.87	4.11E-195
	Robo3	7.75	13.06	0.70	0.000166
	Kdm5d	0.00	9.95	10.38	1.07E-275
	Gli1	4.56	7.92	0.79	7.02E-08
	Syt4	4.12	6.86	0.74	7.17E-07

c

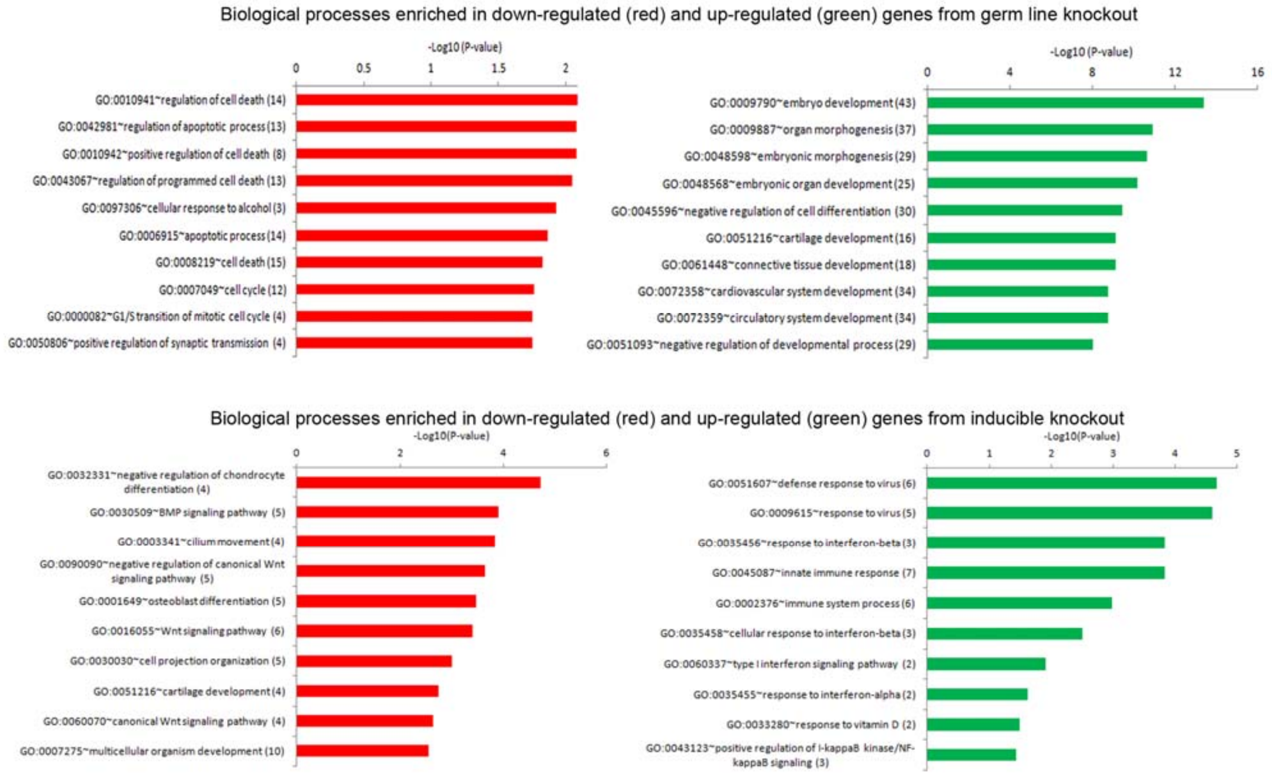

Supplementary Figure 10 Changes of gene expression patterns in the cortex of *Kif20a* knockout mice

a. RNA-seq data were obtained from the E12.5 cortical cells derived from germline or inducible *Kif20a* knockouts. The residue Kif20a RNA-seq signal in the three germline knockouts were located in the exons #1-4 (data not shown), consistent with the knockout-first strategy illustrated in Figure S7a, in which insertion of the LacZ-Neo cassette disrupts the production of Kif20a full length transcript. The three inducible Kif20a knockouts showed significant levels of Kif20a RNA-seq signal and this could be due to varied levels of Cre expression in cortical cells.

b. The RNA-seq data revealed 80 down-regulated genes and 193 up-regulated in the germline mutant brains and 59 down-regulated genes and 47 up-regulated in the inducible knockout brains (Fold change >=1.5; P value <= 0.05). The top few genes of each group were listed.

c. DAVID bioinformatics analysis program was used to identify biological processes that were associated with down- or up-regulated gene groups. The top biological processes of each group were shown. Down-regulation of cell cycle genes and up-regulation of cell differentiation genes in the germline knockouts were consistent with the observation that LOF of KIF20A promoted cell cycle exit and differentiation.

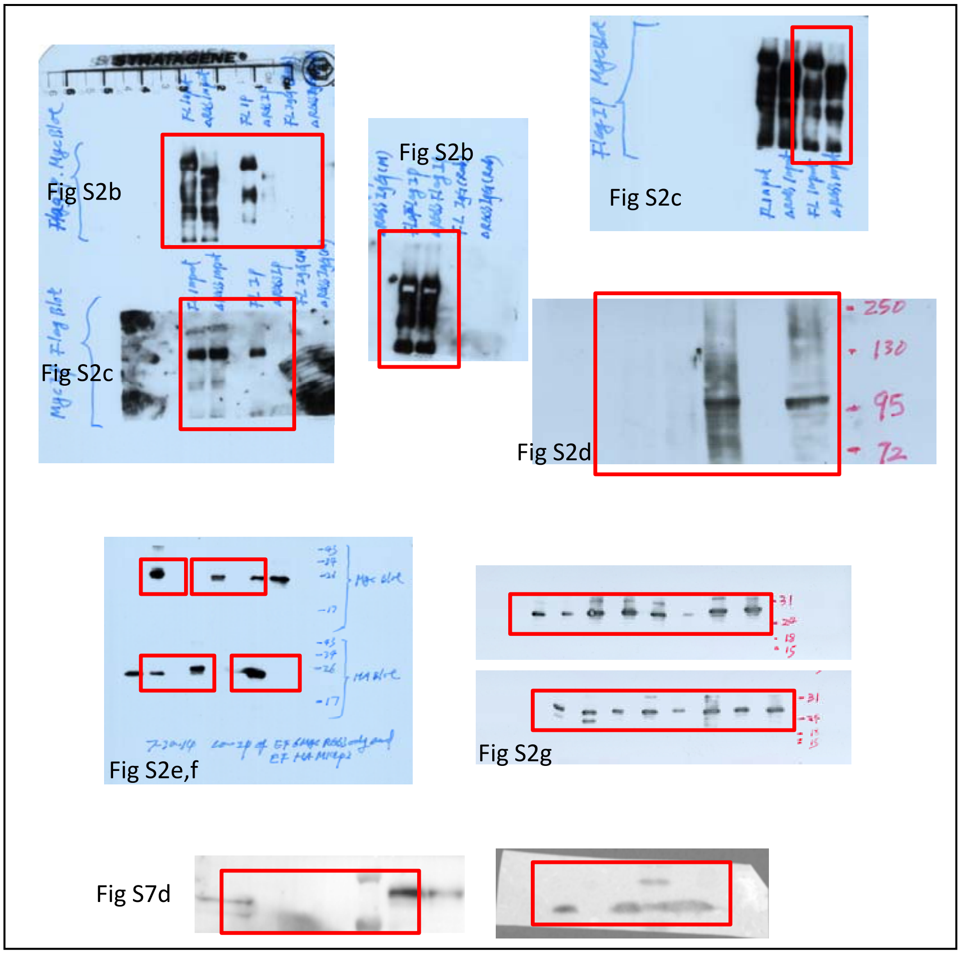

Supplementary Figure 11 Original blots for images used in Supplementary Figure S2b-g and S7d
