# Supplementary material for: KIF20A/MKLP2 regulates the division modes of neural progenitor cells during cortical development
Source: Nat Commun. 2018 Jul 13;9:2707. doi: 10.1038/s41467-018-05152-1 (PMC6045631; doi:10.1038/s41467-018-05152-1)
Supplement: Supplementary file 3 — Description of Additional Supplementary Files [file 41467_2018_5152_MOESM3_ESM.docx]

**Description of Additional Supplementary Files**

File Name: Supplementary Data 1

Description: RNAs obtained from cortical cells of littermates of E12.5 wildtypes and germline Kif20a knockouts were sequenced by deep sequencing. Genes differentially expressed between wild-type and homozygous mutant cortical cells were summarized in this excel file.

File Name: Supplementary Data 2

Description: RNAs obtained from cortical cells of littermates of E12.5 wildtypes and conditional Kif20a knockouts were sequenced by deep sequencing. Tamoxifen was administered at E9.5 and E10.5 consecutively. Genes differentially expressed between wild-type and homozygous conditional knockout cortical cells were summarized in this excel file.

File Name: Supplementary Movie 1

Description: Dissociated cells derived from the E13.5 cortices of the DcxmRFP reporter mice were infected with lentiviruses for expression of control shRNA or shKif20a. Live cell images were taken at 12min intervals for 48 hours. This movie shows images of phase contrast and RFP channels of a progenitor-progenitor division.

File Name: Supplementary Movie 2

Description: This movie shows corresponding images of GFP and RFP channels of movie 1. GFP represents shRNA expression.

File Name: Supplementary Movie 3

Description: Dissociated cells derived from the E13.5 cortices of the DcxmRFP reporter mice were infected with lentiviruses for expression of control shRNA or shKif20a. Live cell images were taken at 12min intervals for 48 hours. This movie shows images of phase contrast and RFP channels of a progenitor-neuron division.

File Name: Supplementary Movie 4

Description: This movie shows corresponding images of GFP and RFP channels of movie 3. GFP represents shRNA expression.

File Name: Supplementary Movie 5

Description: Dissociated cells derived from the E13.5 cortices of the DcxmRFP reporter mice were infected with lentiviruses for expression of control shRNA or shKif20a. Live cell images were taken at 12min intervals for 48 hours. This movie shows images of phase contrast and RFP channels of a neuron-neuron division.

File Name: Supplementary Movie 6

Description: This movie shows corresponding images of GFP and RFP channels of movie 5. GFP represents shRNA expression.
